# Arginylation: a new regulator of mRNA stability and heat stress response

**DOI:** 10.1038/cddis.2016.353

**Published:** 2017-02-09

**Authors:** Kamalakshi Deka, Sougata Saha

**Affiliations:** 1 Department of Molecular Biology and Biotechnology, Tezpur University, Napaam, Assam 784028, India

Cellular stress responses are protective mechanism against diverse environmental and physiological factors, which cause macromolecular damage in cells. Different stress conditions, including heat stress, oxidative stress, and ER stress, are associated with denatured or unfolded proteins, which are recognized by molecular chaperons like heat shock proteins (HSPs). Interaction of unfolded proteins with chaperones lead to induction of expression and recruitment of more molecular chaperons, which reestablish the structural integrity of unfolded proteins. For long time, it was known that the level of cytosolic HSP70 during heat stress was boosted not only by induction of gene expression from the inducible isoforms of *HSP70* genes, but also stabilization of the HSP70 transcripts, so that HSP70 protein expression can be sustained for longer period and at high level.^1^ Thus stabilization of HSP transcripts is vital to mount a potent stress response and cell survival. Few factors that were earlier suggested to participate in HSP70 transcript stability include eEF1A1 and miR-378*.^2, 3^ However, a clear picture about how HSP mRNA is stabilized during heat shock yet to emerge. In our recent work published in *Cell Death Discovery*, we reported that protein arginylation plays an important role in this process as cells devoid of protein arginylation failed to stabilize HSP70 and HSP40 transcripts and became susceptible to heat stress.^4^

Arginylation is a protein modification in which proteins are modified by addition of arginine at the N-terminal amino group or side chains of reactive amino acids by enzyme arginyltransferase (Ate1).^5^ Posttranslationally added arginine can be further modified by methylation leading to a unique double modification on the same site of a protein.^6^ Because of distinct positive charge on arginine side chain, it is thought to change the surface property of a protein and can have diverse functionality from destabilization and degradation of a protein to regulation of protein activity and interaction.^7, 8^ Thus arginylation is emerging as a global regulator of cellular physiology by regulating cell survival to cell death.^9, 10^ Cell survival in stressful conditions mainly depends on stress response pathways and involvement of arginylation has been reported in diverse stress conditions, including nitrosative stress, ER stress, and cytosolic misfolded protein stress. Oxidation of Cys residues upon nitrosative or oxidative stress and subsequent arginylation of oxidized Cys is reported to be a key regulatory mechanism during nitrosative and oxidative stress response.^11^ ER residing proteins and chaperon-like calreticulin and GRP78, and other molecular chaperons namely, chaperonin, HSPA8, ribophorin I, HSP90*β,* and HSP90*α* have been reported as substrate for arginylation.^6, 12^ Although ER stress-induced arginylation of calreticulin helps these protein to dimerize and recruited in stress granules,^13^ cytosolic misfolded protein stress induces arginylation of GRP78 leading to its interaction with autophagic adapters.^14^ Large number of reports indicated importance of arginylation during cellular stress responses. However, our current understanding on this aspect probably is at a nascent stage and requires many more years of work to unfold fully.

Our recent study to understand the role of protein arginylation in heat stress response showed that Ate1 KO mouse embryonic fibroblasts (MEFs) (KO cells) are more susceptible to heat stress compared with its wild type (WT) counterparts, a phenotype that can be rescued by stable expression of Ate1 in KO MEFs. Although at the given heat stress condition WT MEFs were protected, apoptosis was induced in KO MEFs. A loss of mitochondrial membrane integrity was also observed in heat stressed Ate1 KO cells. Gene expression analysis of inducible heat shock proteins, HSP70.1, HSP70.3, and HSP40, showed induction in KO MEFs during shorter period of heat shock. However, expressions of these genes are drastically diminished in KO MEFs upon longer period of heat shock, which were recovered by expression of Ate1-1 in KO cells. This raised the question that, why Ate1 KO cells are not protected in longer period of heat stress in spite of induction of HSPs. Earlier reports indicated that apart from induction of transcription, stabilization of the transcripts are also vital to achieve required protein levels of HSPs during heat stress conditions.^1^ We hypothesized that loss of arginylation may have affected the stability of HSP transcripts thus dampening the stress response leading to induction of apoptosis. When we tested the stability of HSP transcripts in WT and Ate1 KO cells, we found that loss of arginylation (Ate1 KO) indeed reduced the stability of all three HSP mRNAs. Considering this, it is highly possible that KO cells could not accumulate enough HSP proteins to mount a potent stress response due to faster degradation of HSP mRNAs. Apart from protein folding, HSPs also inhibit apoptosis during heat stress by inhibiting release of pro-apoptotic proteins from mitochondria. Taken together these observations, it was proposed that arginylation-dependent stabilization of HSP transcripts help cells to attain high amount of HSP proteins, which not only resolve structural integrity of proteins, but also inhibit apoptosis (Figure 1a). On the other hand, in absence of arginylation, cells fail to stabilize HSP transcripts during stress condition leading to lower amount of HSP proteins, which is insufficient to resolve miss-folded proteins and fails to inhibit apoptosis (Figure 1b).

HSPs play important roles in diverse disease conditions. Although overexpression of HSPs shown to have protective effect in several neurodegenerative disorders, HSP90 is been tested as an anticancer target due to its higher expression in many cancer cells where it help to fold many oncogenic proteins. Identification of arginylation as a regulator of HSP protein expression opens an exciting direction of research to explore its involvement in diverse disease conditions and search for its targets. Another exiting direction of investigation will be newly found function of arginylation in regulating mRNA stability, understanding its mechanism and implications in cellular physiology.

## Figures and Tables

**Figure 1:**
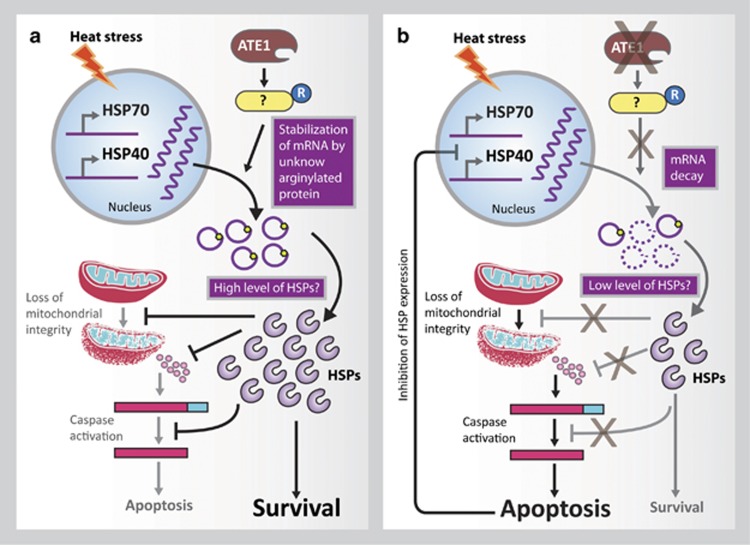
Protein arginylation plays a protective role during heat stress. (**a**) Arginylation of an unknown target facilitates HSP mRNA stabilization leading to sustained expression of HSP proteins and cellular protection by maintaining mitochondrial integrity and inhibition of apoptosis. (**b**) Cells lacking arginylation fails to stabilize HSP mRNA possibly due to absence of arginylation in key protein/s. This causes insufficient level of HSP proteins resulting in weak stress response and cell death

## Abbreviations

HSP: heat shock protein
eEF1A1: eukaryotic elongation factor 1 alpha 1
miR: micro RNA
Ate1: arginyltransferase 1
GRP: glucose regulated protein
MEF: mouse embryonic fibroblast
KO: knock out
WT: wild type
ER: endoplasmic reticulum
